# Ultrasound assessment of gastrointestinal luminal contents: a narrative review

**DOI:** 10.1007/s40477-024-00951-3

**Published:** 2024-09-18

**Authors:** Heidi Y. Su, Kirstin M. Taylor, Antony B. Friedman, Giovanni Cataletti, Giovanni Maconi

**Affiliations:** 1 Department of Gastroenterology, The Alfred Hospital and Monash University, Melbourne, Australia; 2 Gastroenterology Unit, Department of Biomedical and Clinical Sciences, “L. Sacco” Hospital, University of Milano, Milan, Italy

**Keywords:** Ultrasound, Gastro-intestinal, Content, Constipation, Dilatation, Bezoar

## Abstract

Gastro-intestinal ultrasound (GIUS) is a non-invasive and cost-effective tool, widely used as a first-line diagnostic method in patients presenting with abdominal complaints, especially in patients affected by inflammatory bowel disease (IBD), such as Crohn’s disease and ulcerative colitis. In this setting, gastro-intestinal ultrasound has been especially used to evaluate the bowel wall features (thickening, stratification, vascularization) and complications related to IBD (fistulas, abscesses). Nevertheless, gastro-intestinal ultrasound can be also used to detect and evaluate the content of several segments of the gut. In fact, there is a growing interest in utilizing GIUS for suspected functional disorders, where assessing intestinal content may play a significant diagnostic role, as well as directing therapy. In our review, we provided a sonographic description of GIUS appearances of bowel content in various pathological and physiological conditions, offering potential applications in clinical practice and providing insights for further research.

**Supplementary Information:**

The online version contains supplementary material available at 10.1007/s40477-024-00951-3.

## Introduction

Transabdominal gastro-intestinal ultrasound (GIUS) is currently widely used to detect and monitor several acute and chronic gastrointestinal diseases. So far, its diagnostic ability and accuracy rely on proper evaluation of bowel wall features and extraintestinal findings. However, GIUS can also detect and evaluate the content of several segments of the gut, including the oesophagus, stomach, small intestine and colon. The assessment of intestinal contents may provide clues to the diagnosis of certain diseases and conditions. Ultrasound (US) has advantages over other radiological techniques, being cheaper, easily accessible, and radiation-free. It can be used to assess and monitor progress after treatment initiation of specific clinical conditions characterised by abnormalities or changes of gastrointestinal luminal content.

Here we review the reported applications in the scientific literature of GIUS for assessment of gastrointestinal luminal contents, and its practical impact and potential insights on various functional and organic diseases.

## Oesophagus

### Oesophageal food impaction

Point-of-care ultrasound in the emergency department can be used to evaluate oesophageal food impaction. A case series using neck ultrasound was able to confirm ongoing food impaction in the oesophagus, whose cervical tract is visible left to the trachea and left pole of the thyroid, showing dilatation and persistent intraluminal air-fluid levels after swallowing [1].

### Achalasia

**Fig. 1:**
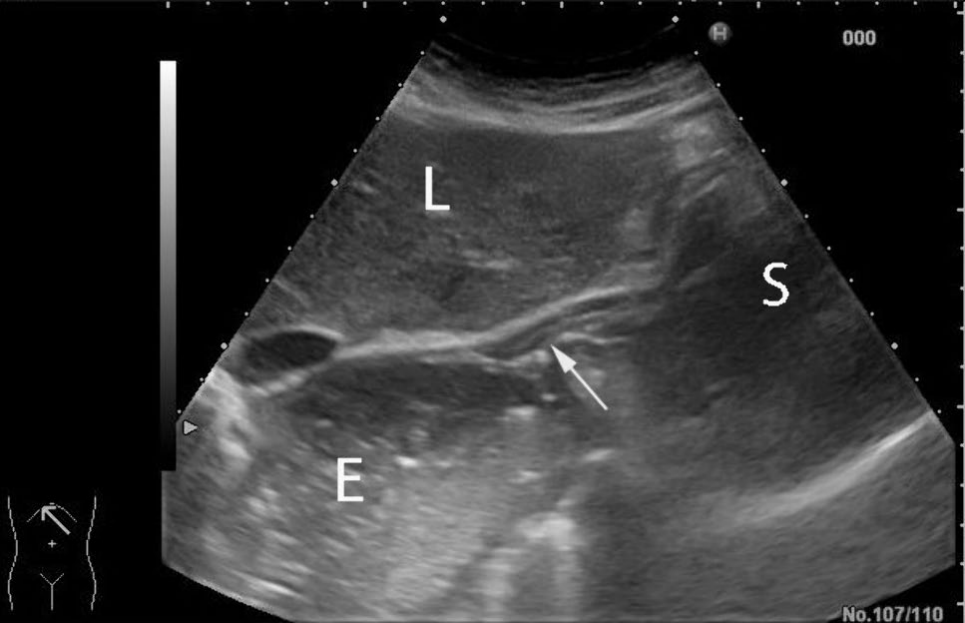
Dilated distal oesophagus with gaseous and fluid content (E) and gastric cardia (arrow) in a patient with achalasia, observed with an oblique subcostal scan; L: Liver; S: Stomach. Videoclip available (videoclip 1)

In achalasia, transabdominal ultrasound has been able to show in more than three quarters of patients significant dilatation of the distal oesophagus with fluid retention. This is noted in both a fasting state and after ingestion of water, along with a regular thickening of the walls of gastric cardia [2] (Fig. 1).

## Stomach

### Assessment of gastric contents before sedation or anaesthesia

There has been great interest over recent years in the utilisation of ultrasound in the assessment of gastric contents before sedation or anaesthesia with regard to pulmonary aspiration risk. Although guidelines exist regarding the duration of restriction of fluid and food intake before sedation or anaesthesia, these apply only to healthy individuals for elective procedures, and may not be reliable in those with co-existing disease or in emergency situations. Ultrasound allows for rapid assessment of gastric volume and discrimination between gaseous, liquid or solid contents [3].

**Fig. 2:**
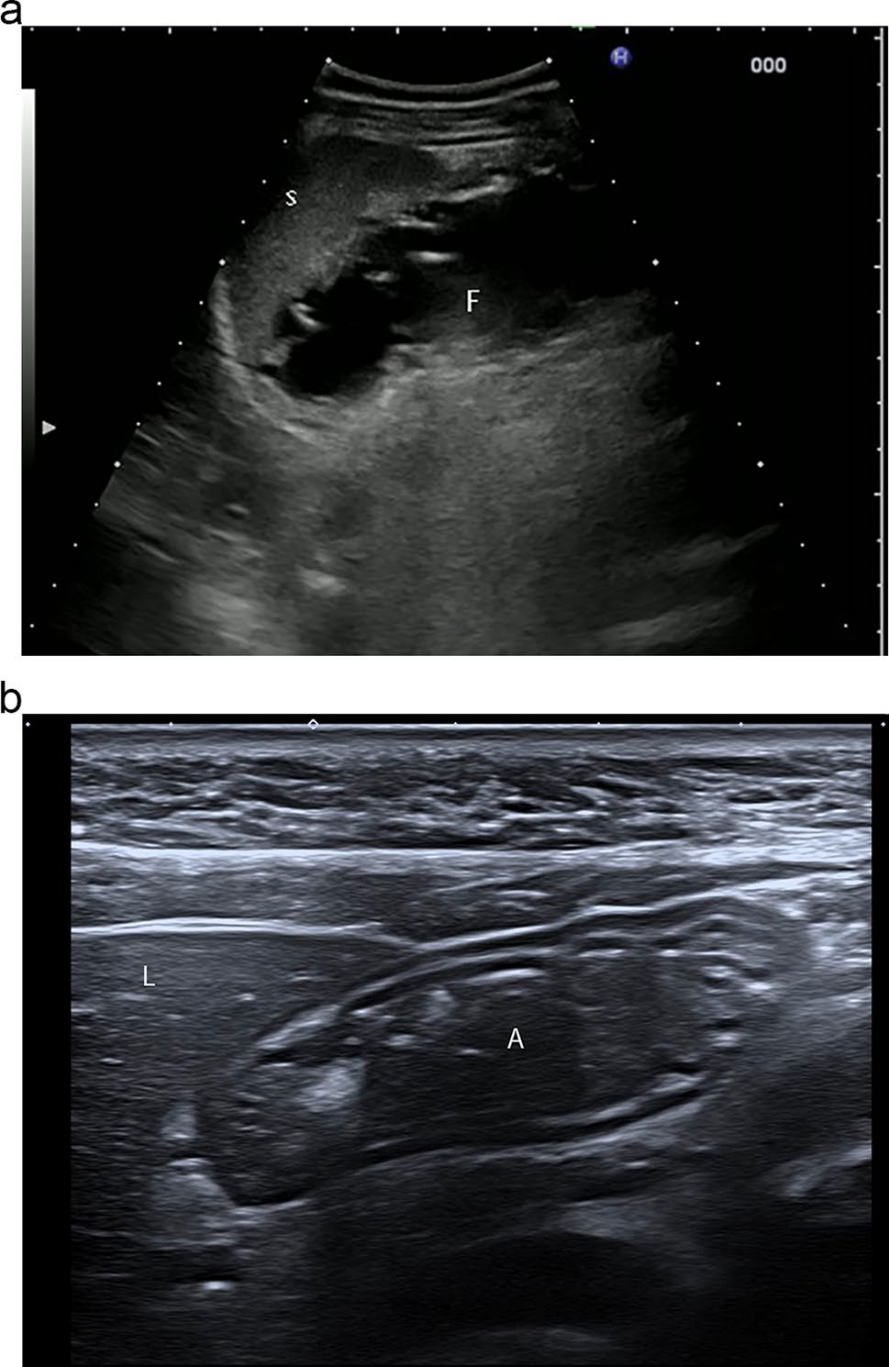
**a** Fluid filled gastric fundus (F) detectable by left transcostal approach in semi left-lateral decubitus, using spleen (S) as acoustic window. **b** Transverse scan of the gastric antrum (A) with small amount of fluid content, appreciable just below the left lobe of the liver (L)

The optimal position of the patient in determining gastric volume depends on which part of the stomach is to be examined. The fundus is often difficult to image due to the deep location and lack of acoustic window. However, it may be seen in a left lateral intercostal, trans-splenic approach, in supine or semi-sitting decubitus position [3] (Fig. 2a). The antrum and body are better evaluated in a semi-sitting or right lateral decubitus position, and these positions are particularly important to estimate lower volumes of gastric fluid [4] (Fig. 2b).

Ultrasound has been used to assess gastric volume in pregnant women, including in the third trimester, following epidural analgesia during labour, before elective caesarean section, and postpartum [5–8].

**Fig. 3:**
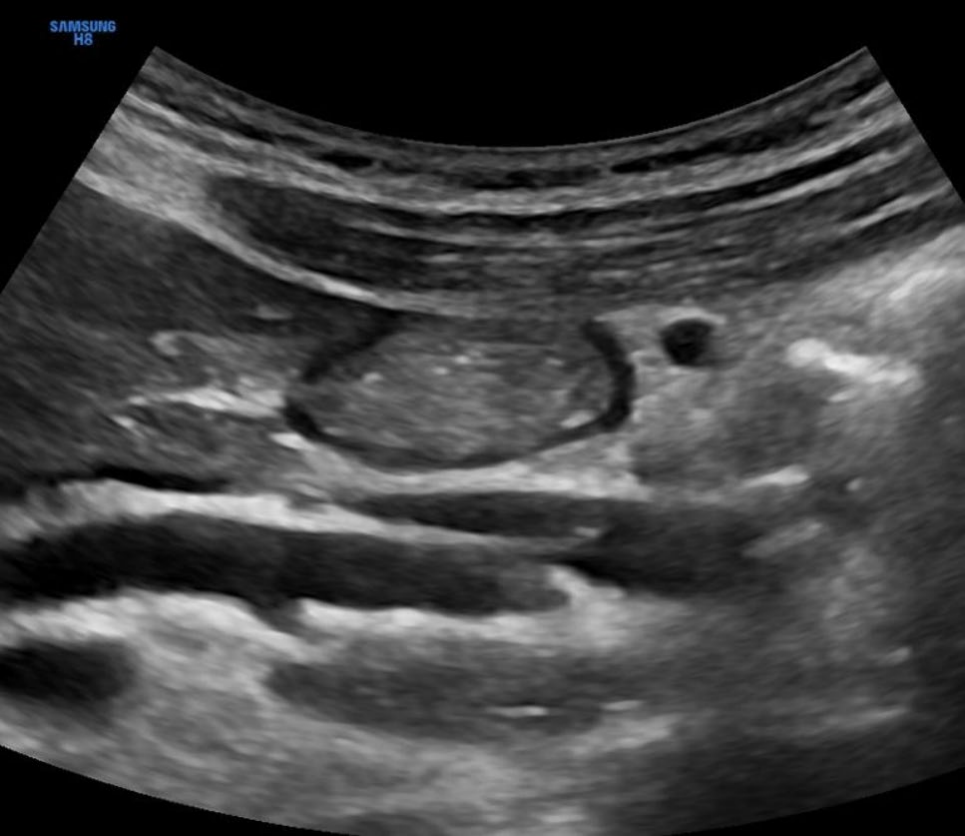
Aspect of gastric antrum after a regular mixed-solid meal

Clear fluids and baseline secretions are hypoechoic or anechoic on ultrasound, and the presence of gas bubbles gives a ‘starry night’ appearance [3, 9]. Thicker fluids have increased echogenicity [10]. Recently chewed solid food has a ring-down artefact and a ‘frosted-glass’ appearance due to the amount of air mixed with the food. Over time, the air separates out and solid content can be seen with a mixed echogenicity [3, 10] (Fig. 3).

Gastric volume can be estimated by measuring the antral cross-sectional area (CSA). Multiple models of varying complexity for assessment of gastric volume have been reported in adults, children, obese people, pregnant women, and critical care patients [4–8, 11–15]. One such formula calculates the elliptical area: CSA = (longitudinal diameter × anteroposterior diameter × π)/4 [9]. A simpler, semi-quantitative grading system has been reported to determine low from high volume gastric contents based on qualitative evaluation of the clear-fluid containing gastric antrum in the supine and right lateral decubitus positions [16]:

- Grade 0—empty in both positions.
- Grade 1—empty in supine but clear fluid visible in right lateral decubitus position.
- Grade 2—clear fluid seen in both positions.

**Fig. 4:**
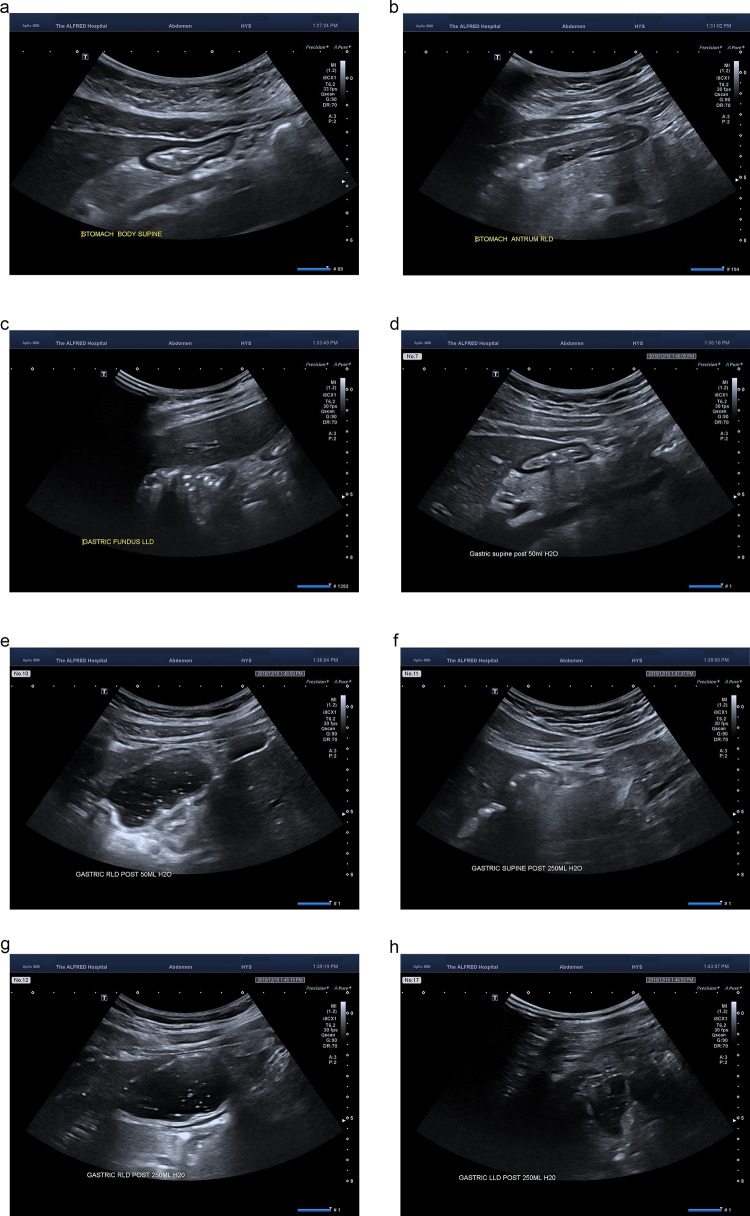
Different gastric volume at different positions **a**–**c** Empty stomach supine/RLD/LLD; **d**, **e** 50 ml water supine/RLD; **f**–**h** 250 ml water supine/RLD/LLD

A subsequent validation study showed that subjects with an antral grade of 1 had under 100 ml of gastric fluid in 75% of cases and those with an antral grade of 2 had over 100 ml of fluid in 75% of cases [17]. The same grading system has also been used in children, obese people and pregnant women [6, 7, 12, 14, 15] (Fig. 4).

### Assessment of gastric emptying and gastric accommodation

Ultrasonographic evaluation of gastric content can be used to assess various gastric functions including antral contractility, gastric emptying, trans-pyloric flow, gastric configuration, intra-gastric distribution of meals, gastric accommodation, and strain measurement of gastric wall in response to liquid test meals [18].

Assessment of gastric emptying with ultrasound has been used for investigation of digestion and satiety, effects of diets, therapeutic agents, and conditions associated with impaired gastric motility [19–22]. US gastric emptying, consisting of serial standard sagittal measurements of the antral area, at the level of aorta and superior mesenteric vein, before and after ingestion of a test meal, has been studied using ultrasound in healthy adults and children, and also in preterm infants and in pregnant women during labour while under epidural analgesia [8, 23–25]. Ultrasound assessment of gastric emptying had been demonstrated to correlate well with scintigraphy [26–28]. Delayed gastric emptying and abnormal antral distension have been noted on ultrasound in adults with diabetes mellitus, cystic fibrosis, systemic scleroderma, systemic lupus erythematosus, acquired immune deficiency syndrome, asthma, Parkinson’s disease, after mental stress, and functional dyspepsia [21, 26, 27, 29–36].

Gastric accommodation (how the stomach compartment changes in response to a meal) has been assessed using ultrasound to measure the size of the proximal stomach with oblique scan of the fundus, after a standardised liquid meal [37]. This is less invasive than the “gold standard” Barostat test. A recent study of over 500 patients with functional gastrointestinal disorders, found a similar prevalence of impaired gastric accommodation using ultrasound (36%), when compared to previous studies using Barostat (40%) [38, 39]. The same group conducted an ultrasound assessment of gastric accommodation in patients with insulin dependent diabetes mellitus with impaired vagal tone, reflux oesophagitis, and children with recurrent abdominal pain. They identified that impaired postprandial gastric meal distribution correlated with patient symptoms [40–42].

## Small intestine

### Small intestine bacterial overgrowth

**Fig. 5:**
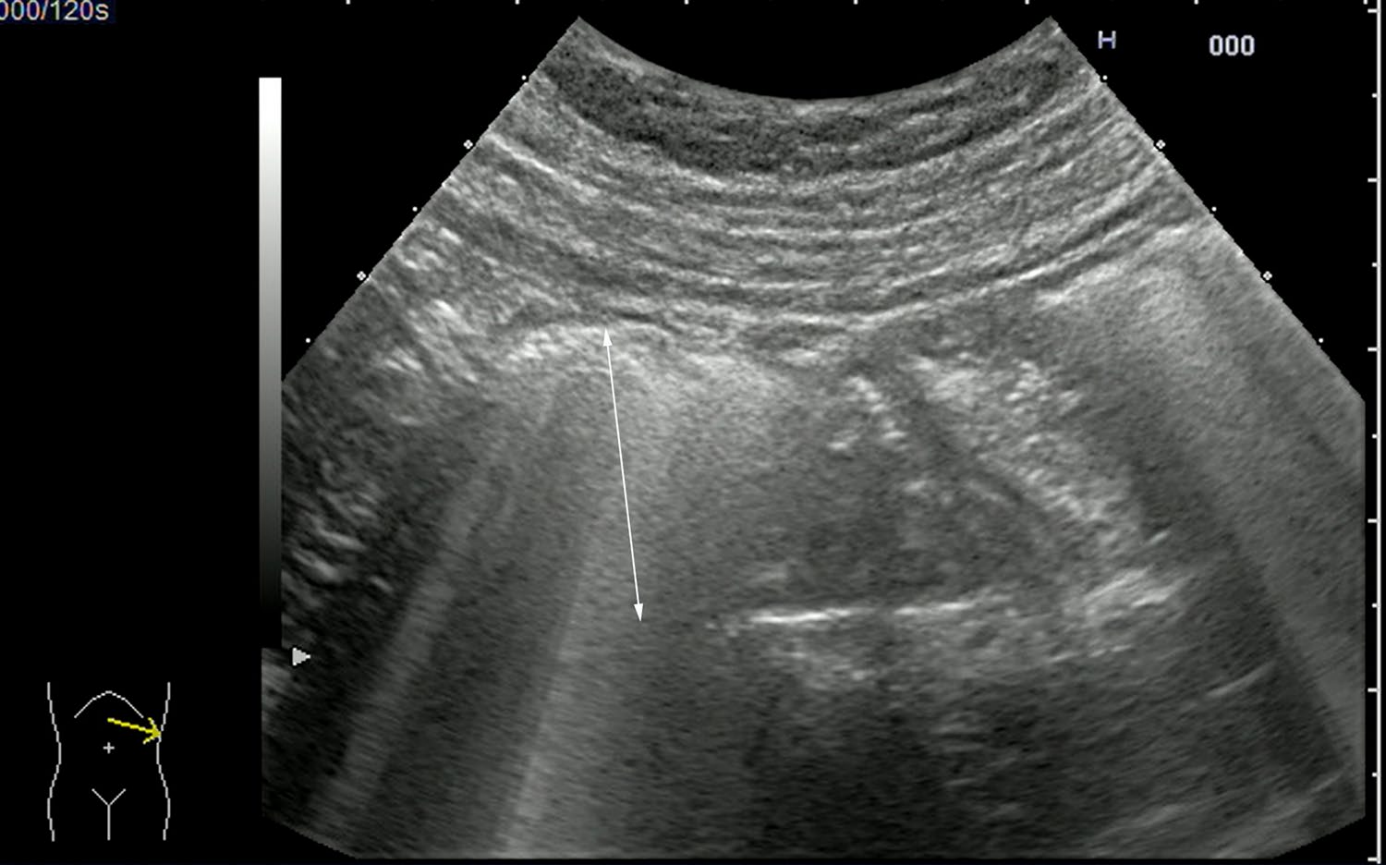
Increased fluid and gas content, along with mild dilatation (arrow) of jejunal loop in a patient with small intestine bacterial overgrowth (SIBO) detected by glucose breath test. Videoclip available (Videoclip 2)

There is limited literature regarding the sonographic findings of small intestine bacterial overgrowth (SIBO). A case series of 176 patients with small intestine pathology included 35 patients with SIBO [43]. Excessive fluid and gas were seen in the small intestinal lumen (Fig. 5). Jejunal fold thickening and increased jejunal motility were also common.

### Small bowel obstruction

Ultrasound can be used to assess suspected small bowel obstruction (SBO), however views can be limited by excessive air. This can be reduced by applying graduated pressure, and by examining all areas, including the flanks. Sonographic findings include: increased luminal fluid and/or gas; dilated loops of small bowel with diameter of over 25 mm; and increased small bowel peristalsis along an intestinal segment longer than 10 cm [44, 45]. The site of obstruction can be defined by assessing the jejunal folds that are often visualized when the level of obstruction is at or just below the jejunum. Conversely, when the obstruction is in the distal small bowel, segments without folds are visible because the folds are less prominent and less frequent in the ileum. Distal to the level of obstruction, the small bowel and colon are empty and collapsed. Free fluid can be seen between the dilated small bowel loops, and is often associated with worsening mechanical obstruction and need for surgery [46]. A case series of 123 patients found an accuracy of sonographic diagnosis of small bowel obstruction of 81.3% overall, and 91.7% after excluding 14 cases with excessive intestinal gas [44]. The level of SBO was identified in 80.5%, by incorporating the location of the dilated small bowel loops and the intestinal fold pattern. The cause of obstruction was diagnosed on ultrasound in 62.6% cases of mechanical obstruction and 40.6% cases of small bowel ileus.

**Fig. 6:**
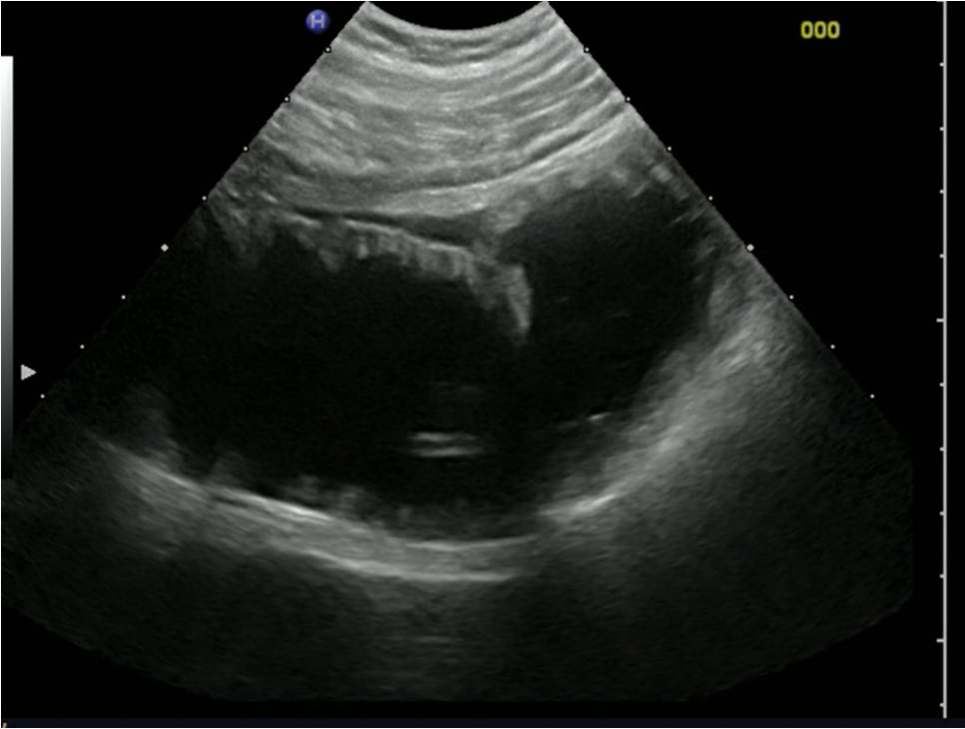
Image of paralytic ileus in a patient with acute appendicitis and peritonitis showing a dilated jejunum (> 3 cm) with clear fluid content and without peristaltic activity of contractions, over a length > 10 cm. Videoclip available (videoclip 3)

Paralytic ileus should be considered when dilated small bowel loops are seen that are filled with fluid and debris, yet no peristaltic movement is identified [44]. There is a lack of a transition point. The colon is usually filled with fluid, gas, and stool (Fig. 6).

Gallstone ileus can present as a curvilinear, echogenic structure with a well-defined shadow, associated with a dilated, fluid filled bowel loop. These findings are similar to those seen with bezoars, however, gallstone ileus may be suspected when air is seen in a thick-walled and often collapsed gallbladder [47, 48]. Overall, CT is more sensitive than ultrasound for diagnosis of gallstone ileus, and more accurate to identify the level and causes of obstruction and complications such as bowel wall oedema, ischaemia, perforation, and biliary intestinal fistula [49]. However, CT requires radiation and the use of intravenous contrast, and CT identification of gallstones may be limited by the composition and structure of the stones.

In addition, in patients with suspected small bowel obstruction, GIUS, also used as point-of-care ultrasonography has a high accuracy in diagnosing small bowel obstruction when performed by trained physicians, and may substantially decrease the time to diagnosis, and speed up or avoid CT scans [45, 50, 51].

## Colon

### Constipation

**Fig. 7:**
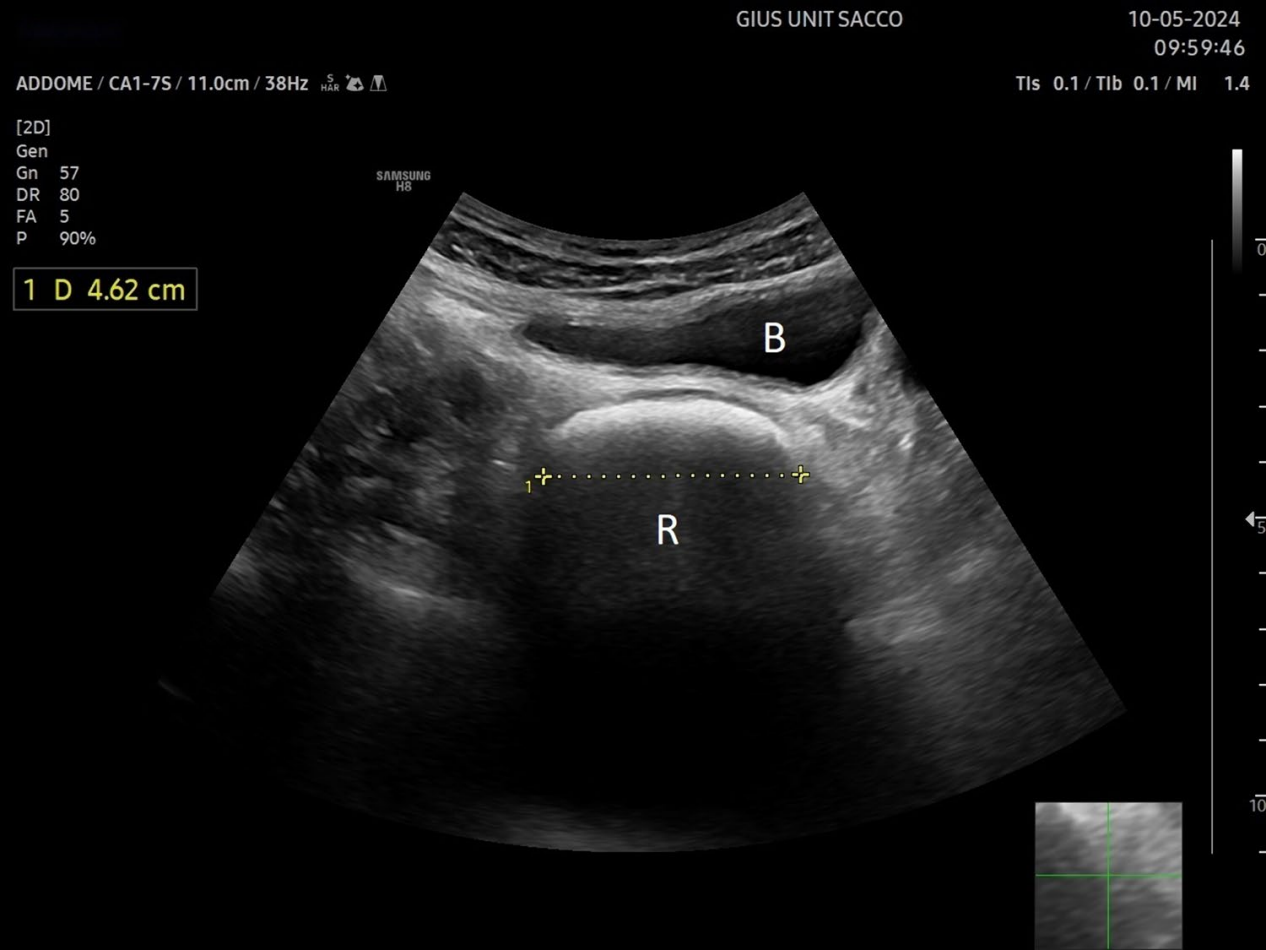
Transverse scan of the rectum. US exam showed, behind the urinary bladder (B), a dilated rectum (R) containing solid stools

Ultrasound measurement of rectal diameter has been used for many years in children and adults to assess constipation and faecal loading in the rectum (Fig. 7). In children, the accuracy of ultrasound as a predictor of constipation and faecal impaction is still controversial [52, 53]. Rectal diameter alone does not provide information about the extent of faecal loading and the consistency of the stool. The rectopelvic ratio, namely the ratio of the width of the rectum assessed by ultrasound to the distance between the anterior superior iliac spines (measured using a tape), of greater than 0.189, allows the detection of megarectum with faecal impaction with a sensitivity of 88.3% in children with functional chronic constipation [54].

A scoring system was developed that takes into account the height of the stool (nil/behind/above bladder, nearly at/at/above umbilicus, upper edge not seen) and the effect upon the bladder (nil, indented, flattened, displaced) [55]. The ultrasound score showed good correlation with the symptom score in large cohort of 500 children. A subsequent study of 107 children was unable to validate the score. However, this was only a retrospective review of prospectively collected data [56].

A study of 43 adult patients with evidence of faecal loading on CT showed that ultrasound could also be used to qualitatively assess this [57]. Ultrasound images were allocated as faecal loading detected as:

1. Crescent-shaped acoustic shadows with haustrations.
2. No haustrations with strong high echoes of the colonic lumen.
3. No haustrations with weak high echoes of the colonic lumen (the weak high echoes correspond to higher colon-gas ratio, indicating softer stool consistency).

A modified version was subsequently utilised in a study of 95 elderly patients with physical and cognitive impairment living in long term care facilities. Ultrasound was able to identify the faecal distribution changes in the colon and the rectum [58]. The findings correlated well with the bowel frequency and stool consistency.

Another study compared 268 adult patients with chronic constipation with 66 age and sex matched healthy controls using a “constipation index” based on the average transverse diameters of the five different segments of the colon (ascending, transverse, descending, sigmoid, and rectum) [59]. The ultrasound findings correlated well with the CT findings of stool and/or gas distribution, and also had positive correlation with the colonic transit time as evaluated by radiopaque markers. The constipation index was significantly higher in those with chronic constipation than in healthy subjects, although the cut-off was not clearly defined, which may limit the clinical application of this index.

### Faecal impaction and faecoliths

**Fig. 8:**
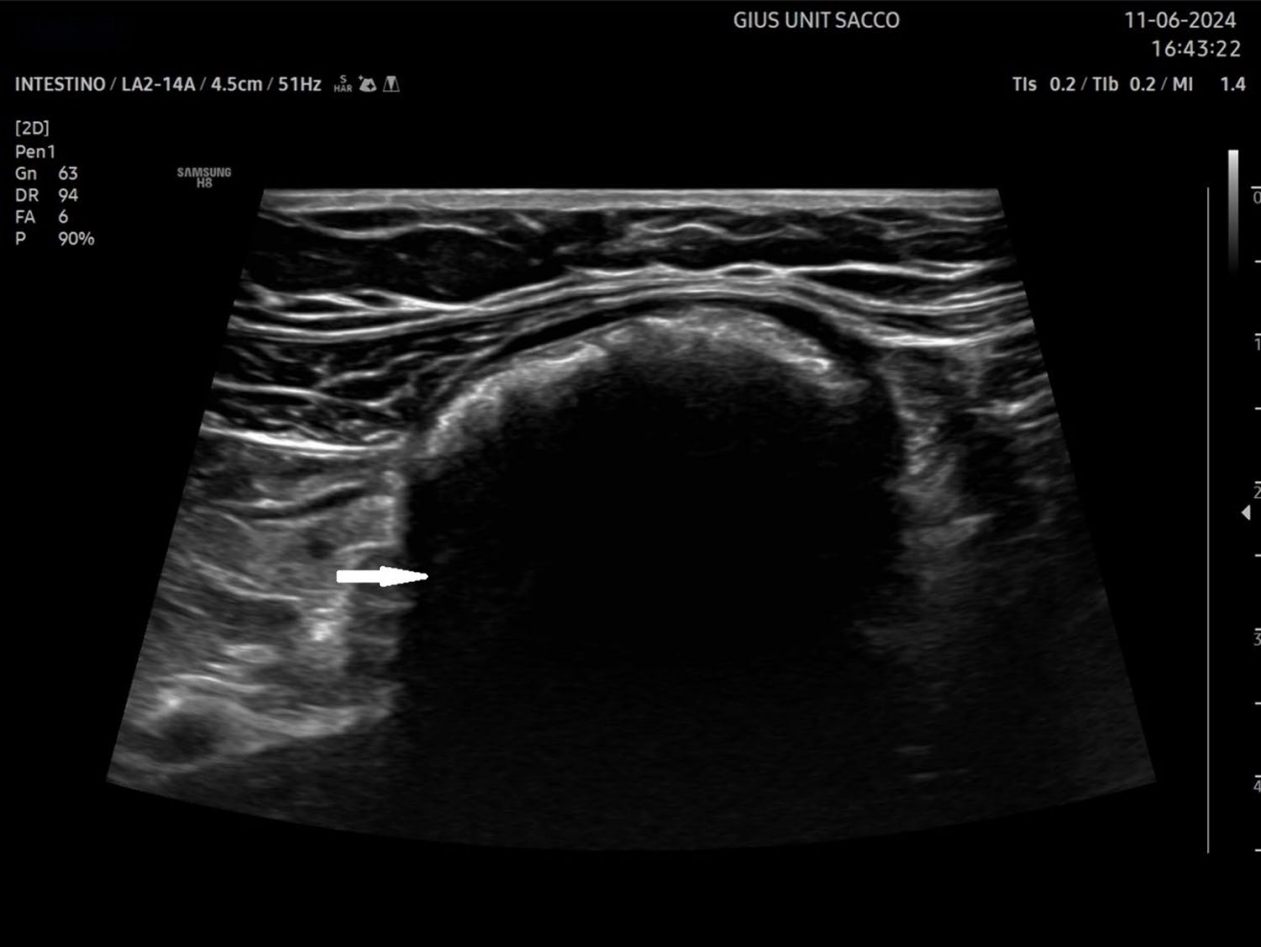
Faecolith in the distal sigmoid colon with a highly echogenic surface and posterior acoustic shadow (arrow), suggesting hard, dehydrated faecal matter

Faecal impaction can result in a faecolith (hard, dehydrated faecal matter) in the rectum or sigmoid colon with sonographic appearances of a pelvic mass with a highly echogenic surface and a posterior acoustic shadow (Fig. 8) [60]. Caecal faecoliths have a similar sonographic appearance in the right iliac fossa and usually occur in older women with chronic constipation [61].

A faecally impacted appendix can be misdiagnosed as appendicitis. A study of 160 patients who underwent ultrasonography for right iliac fossa pain found 22 cases of a normal but faecally impacted appendix [62]. The criteria used to distinguish a faecally impacted appendix from acute appendicitis included:

1. The preservation of the normal layering of the appendiceal wall.
2. Smaller maximal outer diameter.
3. Thinner maximal mural thickness.
4. The absence of peri-appendiceal mesenteric infiltration.
5. No increased blood flow in the appendiceal wall.

**Fig. 9:**
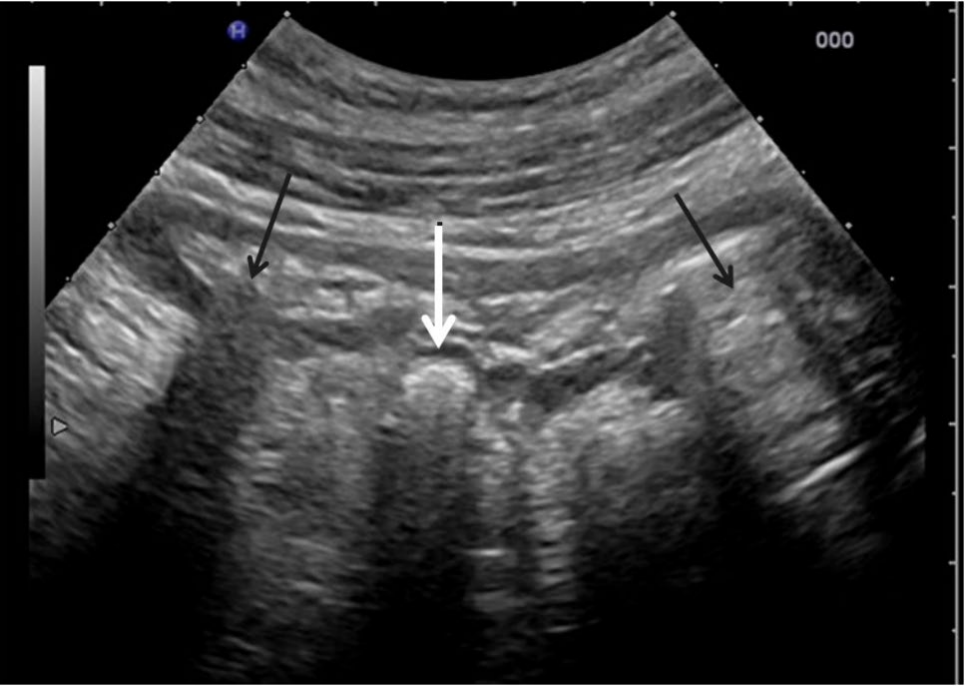
Small faecolith seen within a diverticulum of the left colon. Note the posterior shadowing effect (white central arrow) originating from a diverticular structure outside of the bowel wall, similar to that of an another faecolith observed within the lumen (left arrow) but clearly different from that the ring-down artefact, originating from gas content into the lumen, detected on the right (right arrow)

In colonic diverticulosis, faecoliths may be seen within diverticula. (Fig. 9) In the follow up of acute diverticulitis, in ongoing resolving cases, ultrasound may show evacuation of the faecolith into the colonic lumen, and an empty diverticulum may be seen [63].

## General gastrointestinal tract

### Bezoars

**Fig. 10:**
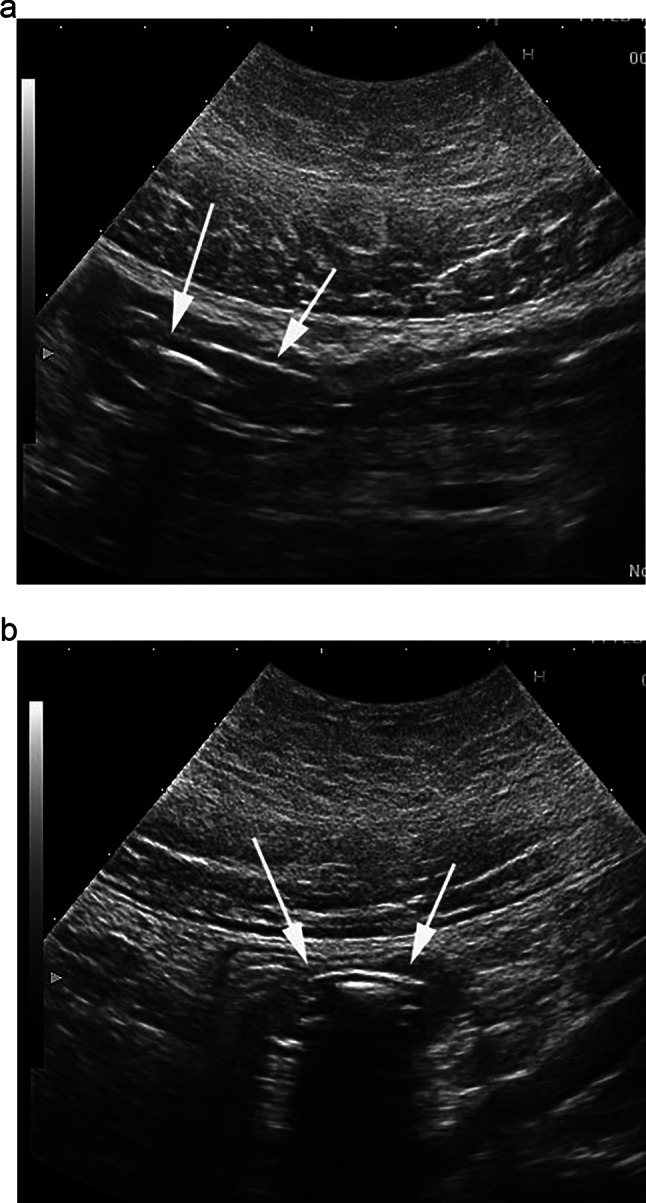
Longitudinal (**a**) and transverse (**b**) scans of the distal small bowel containing undigested petals of an artichoke (arrow)

**Fig. 11:**
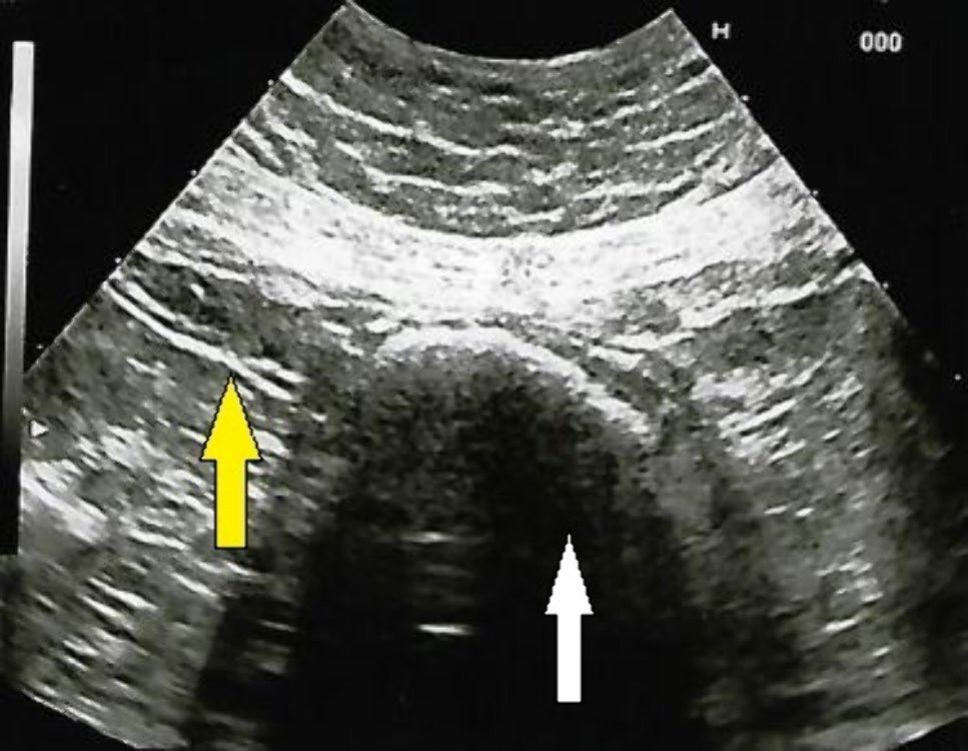
Transverse scan of small bowel containing phytobezoar (white arrow) just upstream a stricture (yellow arrow) in a patient with Crohn’s disease of the ileum subjected to previous stricturoplasty

Bezoars, including trichobezoars (hair) and phytobezoars (plant material), usually form in the stomach and can pass into the small bowel. Rarely, they can cause small bowel obstruction, and even gastric outlet obstruction. The diagnosis of bezoar is important, as its management often involves surgery. CT is the most common imaging modality, where the bezoars usually appear as well-defined, oval, mottled gas-patterned, faeces-like mass, with associated proximal dilatation [64]. On ultrasound, bezoars often appear as hyperechoic, arc-like surfaced masses with a posterior acoustic shadowing [65]. A twinkling artefact on colour Doppler may also be seen, and can help to differentiate bezoars from a faecal mass [66]. A target lesion with a hyperechoic centre within a hypoechoic mass has also been reported [66, 67]. A retrospective study comparing CT to ultrasound, found CT to be more sensitive for diagnosing bezoars, particularly gastric and multiple bezoars [64]. However, CT appearance of bezoars can be similar to food or faecal material. Ultrasound may help to enhance CT findings and confirm the diagnosis, and may also detect potentially dangerous undigested vegetables in the lumen like artichokes and persimmons (Figs. 10, 11) which may form phytobezoars [68, 69]. In a series of 14 patients who presented with small bowel obstruction and suspected bezoars on CT, subsequent ultrasound was able to accurately diagnose the bezoars in 7 patients, which were then confirmed at surgery [70].

### Foreign bodies

Ultrasound has been used for assessment of ingested foreign bodies within the gastrointestinal tract. It can be used as an alternative to, or to augment, x-ray or CT. Its use is not limited to radiopaque objects and may avoid unnecessary radiation exposure. Case reports show ultrasound is able to accurately identify foreign bodies in the oesophagus, stomach, small intestine, colon, rectum, and even in the appendix [71–75]. Ultrasound is valuable for management, particularly in children, and can be used to follow up the progression of foreign bodies, and for planning endoscopy or surgery. It can also be used to assess complications such as obstruction and perforation.

Assessment of foreign bodies in the stomach can be enhanced by placing the patient in the right lateral decubitus position, and with adequate distension of the stomach with fluid such as water, which can displace gas and provide an acoustic window [76].

Most foreign objects are hyperechoic on ultrasound and often produce posterior shadowing. The appearance varies depending on the composition of the object [77]. Metal and glass objects often produce a posterior shadow with reverberation artefact (ring-down or comet-tail artefact), particularly those with a smooth surface [78, 79]. Wood, plastic, and stone objects usually produce posterior shadowing. A more heterogeneous shadowing (“dirty shadowing”) may occur if there is air content in the wooden objects [80]. As an example, a toothpick appears as a hyperechoic, thin, straight line with variable shadowing in longitudinal view, and as a hyperechoic dot with sharp posterior shadowing in transverse view [81]. Measurement of the toothpick length may be underestimated, particularly if it is partly lodged in the wall of the gastrointestinal tract.

### Nasogastric/nasojejunal tube placement

Ultrasound can be used as an alternative to x-ray for assessment of nasogastric and nasojejunal feeding tube position [82, 83]. In an intensive care unit, ultrasound was able to accurately locate the nasogastric tube tip in 34 out of 35 procedures, using x-ray as a comparator [82]. This is a rapid and convenient test that can also be applied to those with contraindications to radiation exposure, such as pregnant women.

### Assessment of ingested pills in massive overdose

Several studies have explored the use of ultrasound for assessing the presence of ingested medication (usually tablets) in the stomach [84]. It was proposed that point-of-care ultrasound in the emergency setting may help with the management of massive overdose, and identifying those who may be suitable for decontamination therapy. Most of the studies were done in simulated conditions, either in vitro with sealed bag of fluid and tablets, or in healthy volunteers after fasting [85–87]. Despite the controlled situations, the ability to detect the presence and quantity of medication was inconsistent. This is likely to be even more difficult in real life settings in non-fasted subjects. Therefore, ultrasound should not be considered a reliable investigation for assessment of ingested medication in the stomach.

### Drug packing

X-ray and CT are the usual mode of imaging for assessments of narcotic drug packets in drug couriers. Ultrasound may be useful as the initial imaging and/or for follow up. Ultrasound has been utilised at airport customs using a portable scanner [88]. A study of 45 cases compared ultrasound findings with CT and found a high sensitivity but a low specificity in suspected cases of both solid and liquid drugs [89]. Solid and liquid drug packets appeared as multiple, immobile, hyperechogenic structures with clean posterior acoustic shadowing. The contour of solid packets is usually smooth, while liquid drug packets often appear irregular. The view of the packets can be enhanced with water ingestion. It is easier to identify packets in fluid filled stomach, caecum, or left colon. Complications such as perforation and obstruction may also be seen on ultrasound [90]. However, a negative ultrasound cannot rule out the presence of drug packets, false positives are also possible due to similar appearances with stool, and ultrasound cannot accurately quantify the number of packets.

### Parasites

Roundworm or *Ascaris lumbricoides* is prevalent in tropical and subtropical regions. It is caused by ingestion of contaminated food, water, or soil, and can cause obstruction of small bowel, biliary tree, pancreatic ducts, and appendix. An adult *Ascaris lumbricodes* worm is typically 15–35 cm long and 2–6 mm thick. Ultrasound has been used to confirm the presence of *Ascaris lumbricoides* in the gastrointestinal tract [91, 92]. The sonographic features are varied, depending on the part of the worm imaged, and whether the worm is alive or dead. The body of the worm usually appears as an echogenic non-shadowing, tubular structure, with a hypoechoic interior, often resulting in 3–4 linear echogenic interfaces. As the worm ingests food, its alimentary tract may turn temporarily echogenic, before becoming anechoic again. This wave of echogenic fluid during the swallow can help to identify the head of the worm. In the transverse section, it is round, and may appear as a target sign or pseudotumour. Serpentine movements can be evoked with probe pressure. *Ascaris lumbricodes* often reside in the jejunum, therefore detection and visualisation may improve in the left lateral decubitus position after ingestion of water [92].

Ultrasound detection of tapeworms (taenia) in the gastrointestinal tract has also been reported. A man presented with abdominal pain was found to have ileal intussusception associated with *Taenia saginata*, seen as a double-reflective, ribbon-like structure in the lumen [93]. Following detection of intestinal taeniasis, ultrasound may help to assess the more serious extraintestinal infestations of cysticercosis, including infestation of the liver, brain, and other soft tissue [94].

### Location of retained capsules/patency capsules

**Fig. 12:**
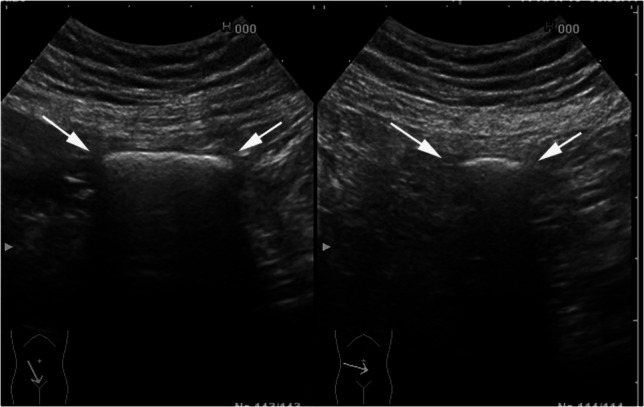
Longitudinal (left panel) and transversal scans (right panel) of a pillcam video endoscopy capsule (arrows) entrapped in the small bowel of a segmental narrowing in a Crohn’s disease patient

The location of retained video endoscopy capsules or patency capsules can be difficult to identify, even on abdominal x-ray. These can be seen on ultrasound as a hyperechoic object with an acoustic shadow (Fig. 12) [95, 96]. In a prospective study of 52 patients with known or suspected small bowel strictures, ultrasound was able to accurately identify the location of the 17 retained patency capsules. Those patients whose patency capsules were seen distal to their small bowel strictures had successful passage of their subsequent endoscopy capsule [97]. In a recent pilot study of gas sensing capsules, ultrasound was used to confirm the location of the capsule as it progressed through the gastrointestinal tract [98].

## Conclusion

Ultrasound is a useful imaging modality for assessing gastrointestinal luminal contents. While most reviews have assessed the role of GIUS in the detection of gastrointestinal diseases, focusing the attention on the sonographic detection and features of intestinal walls, this review has shown that the qualitative and quantitative evaluation of the intestinal content could help in assessing specific gastrointestinal disorders, including functional diseases. The scientific data collected in this review show that sonographic assessment of bowel content is possible and can add useful information to be considered in our clinical practice. These data also represent an important and still not fully explored field for future research.

## Supplementary Information

Below is the link to the electronic supplementary material.

Supplementary file1 (MP4 1739 KB)

Supplementary file2 (MP4 4764 KB)

Supplementary file3 (MP4 2240 KB)

## Data Availability

Data availability is not pertinent for this narrative review article.
